# Reconfigurations of Dynamic Functional Network Connectivity in Large-scale Brain Network after Prolonged Abstinence in Heroin Users

**DOI:** 10.2174/1570159X21666221129105408

**Published:** 2023-01-13

**Authors:** Shan Zhang, Wenhan Yang, Minpeng Li, Xinwen Wen, Ziqiang Shao, Jun Li, Jixin Liu, Jun Zhang, Dahua Yu, Jun Liu, Kai Yuan

**Affiliations:** 1 Center for Brain Imaging, School of Life Science and Technology, Xidian University, Xi'an, Shaanxi, 710126, China;; 2 Engineering Research Center of Molecular and Neuro Imaging Ministry of Education, Xi'an, Shaanxi, 710071, China;; 3 Department of Radiology, Second Xiangya Hospital, Central South University, Changsha, 410011, China;; 4 Hunan Judicial Police Academy, Changsha, 410000, China;; 5 Information Processing Laboratory, School of Information Engineering, Inner Mongolia University of Science and Technology, Baotou, Inner Mongolia, 014010, China;; 6 International Joint Research Center for Advanced Medical Imaging and Intelligent Diagnosis and Treatment & Xi'an Key Laboratory of Intelligent Sensing and Regulation of Trans-Scale Life Information, School of Life Science and Technology, Xidian University, Xi'an, Shaanxi, 710126, China

**Keywords:** Heroin, dFNC, prolonged abstinence, brain network reconfiguration, substance use disorders, neurobiology

## Abstract

**Background:**

Brain recovery phenomenon after long-term abstinence had been reported in substance use disorders. Yet, few longitudinal studies have been conducted to observe the abnormal dynamic functional connectivity (dFNC) of large-scale brain networks and recovery after prolonged abstinence in heroin users.

**Objective:**

The current study will explore the brain network dynamic connection reconfigurations after prolonged abstinence in heroin users (HUs).

**Methods:**

The 10-month longitudinal design was carried out for 40 HUs. The 40 healthy controls (HCs) were also enrolled. Group independent component analysis (GICA) and dFNC analysis were employed to detect the different dFNC patterns of addiction-related ICNs between HUs and HCs. The temporal properties and the graph-theoretical properties were calculated. Whether the abnormalities would be reconfigured in HUs after prolonged abstinence was then investigated.

**Results:**

Based on eight functional networks extracted from GICA, four states were identified by the dFNC analysis. Lower mean dwell time and fraction rate in state4 were found for HUs, which were increased toward HCs after prolonged abstinence. In this state, HUs at baseline showed higher dFNC of RECN-aSN, aSN- aSN and dDMN-pSN, which decreased after protracted abstinence. A similar recovery phenomenon was found for the global efficiency and path length in abstinence HUs. Mean while, the abnormal dFNC strength was correlated with craving both at baseline and after abstinence.

**Conclusion:**

Our longitudinal study observed the large-scale brain network reconfiguration from the dynamic perspective in HUs after prolonged abstinence and improved the understanding of the neurobiology of prolonged abstinence in HUs.

## 1 INTRODUCTION

The human brain is a complex patchwork of interconnected regions, and understanding how the human brain produces cognition depends on knowledge of the configurations among large-scale networks [1]. Addiction is a brain disease associated with abnormal interactions of different brain networks [2, 3]. Four circuits are considered to be the “core” of addiction: reward circuits (saliency network (SN) and basal ganglia network (BG)), inhibitory control/executive circuits (executive control network (ECN)), conditioning/working memory (default mode network (DMN)), and motivation/ drive (sensorimotor network (SMN)) [4, 5]. Recent studies had revealed the functional disruptions of these networks in heroin addiction, such as stronger connectivity between SN and ECN during resting-state, and stronger activation in the DMN when exposed to the heroin-related cue. Meanwhile, the altered connections among key nodes distributed in the SN, ECN, and DMN were correlated with craving or duration of heroin use [6-9]. Besides, similar dysregulated interactions were also in nicotine addiction. Smokers showed weaker SN-ECN and SN-DMN coupling during abstinence, which led to decreased ECN operations and enhanced DMN activity. The combination of decreased ECN activity and less suppression of DMN activity was considered to result in cognitive deficits and difficulty to resist urges to smoke [10, 11]. Taken together, it is speculated that SN may play a causal role in switching between the ECN and DMN, by integrating the processed internal sensory information to regulate the distribution of attention, while other networks regulate reward and cognitive function through the signals sent by SN [5, 12]. Although great progress had been made, abnormal functional connections in these networks in heroin users (HUs) were very limited. Moreover, brain regional and circuit-level recovery after prolonged abstinence had been observed in previous addiction studies [9, 13]. Less is known about whether the brain recovery phenomenon in large-scale brain networks after prolonged abstinence would be detected. Thus, in the current study, we conducted a nearly 10-month longitudinal follow-up tracking on HUs to explore the potential network functional connectivity reconfiguration after prolonged abstinence.

The fMRI resting-state static functional connectivity has been widely used to explore the internal connection of brain networks. However, recent studies have found that the temporal organization of dynamic resting-state functional connectivity patterns follows a specific sequential order in conscious rats and humans by exploring the neuronal origin of dynamic functional network connectivity [14, 15]. Therefore, the brain network resting-state functional connectivity (RSFC) may have several repeated states in the whole fMRI scan, which challenges the conventional assumption that functional interactions remain constant throughout the entire resting-state scan [16]. Additionally, the time-varying characteristic of brain activity was described in neurological and psychiatric diseases such as chorionic low back pain, obesity, migraine and some other diseases [17-19]. It is expected that this will be a particularly dynamic process to test the connectivity changes in the brain of heroin patients. However, few people paid attention to this dynamic change in previous studies. These dynamic functional network connectivity (dFNC) studies suggested the importance of such time-varying characteristics that may represent spontaneous alterations in the large-scale brain networks and provide novel insights into the brain network dynamic reconfigurations derived from the prolonged abstinence in HUs.

In this study, we examined the different dFNC patterns between 40 HUs and 40 healthy controls (HCs) at baseline. Then, we conducted a nearly 10-month longitudinal study to observe whether the abnormal dFNC of brain networks would be reconfigured in HUs after prolonged abstinence. We also conducted topologic and correlation analysis to capture their relationships with clinical symptoms in HUs at both baselines and after prolonged abstinence. We hypothesized that after prolonged abstinence, the HUs showed potential brain network reconfiguration, mainly in the unifying triple networks (SN, DMN, and ECN), which play an important role in addiction. And the dynamic connectivity changes of these networks would be associated with clinical symptom improvement. It is hoped that the current study will improve our understanding of the neurobiology of prolonged abstinence in heroin from the dFNC perspective and provide new evidence for the brain network dynamic connection reconfigurations after prolonged abstinence in HUs.

## 2 MATERIALS AND METHODS

### 2.1 Ethics Statement

Our study was approved by the local Institutional Review Board (IRB) of the Second Xiangya Hospital, Central South University (No. 8167071216), and followed the provisions of the declaration of Helsinki of the World Medical Association.

### 2.2 Participants

Forty abstinence HUs (age: 41.60 ± 6.43 years, male: 28 (70%)) and 40 age-, gender-matched HCs (age: 40.80 ± 9.47 years, male: 26 (65%)) were recruited. Detailed demographic and clinical characteristics can be found in Table **1**. The screening criteria of HUs were as follows: (1) heroin dependence in the fifth edition of the Diagnostic and Statistical Manual on Mental Disorders (DSM-V); (2) short-term abstinence (withdrawal days ≤ 180 days); (3) no history of other drug addiction except heroin and nicotine. The exclusion criteria for both groups included: (1) any physical illness, such as a brain tumor, or obstructive lung disease, as assessed by clinical evaluations and medical records; (2) any current medications that may affect cognitive function; (3) existence of neurological disease; and (4) claustrophobia. All participants were right-handed as measured by the Edinburgh Handedness Inventory.

The first MRI sessions were conducted at baseline, and a follow-up interview was conducted approximately 10 months later to invite HUs to participate in the second MRI scanning. During the 10 months, the patients were admitted to the rehabilitation centers and treated with education and physical exercise; methadone maintenance treatment was not used. The nearly 10-month follow-up study was used to assess the brain network reconfiguration after prolonged abstinence. The visual analog scale (VAS) was used to estimate subjective heroin craving changes in abstinent HUs [20, 21].

### 2.3 MR Imaging Acquisition

MRI data were acquired on a Siemens 3.0T Skyra MRI scanner (Magnetom Skyra, Siemens, Germany) with a 32-channel head coil at the Second Xiangya Hospital of Central South University, Changsha, China. During the MRI scan, participants were asked to keep their eyes open and remain still. To prevent head movement, their heads are limited by a pad and head restraint belt during scanning.

T1-weighted three-dimensional magnetization-prepared rapid gradient echo imaging (3D MPRAGE) was used to acquire the T1-weighted data sets, and the parameters were the following: flip angle = 30°; slices = 176; field of view (FOV) = 256 mm × 256 mm; slice thickness = 1 mm; repetition time (TR) = 1450 ms; echo time (TE) = 2.0 ms; voxel size = 1 mm × 1 mm × 1 mm.

Resting-state fMRI images were acquired with the following parameters: 36 axial slices, thickness = 4 mm, FOV = 220 mm × 220 mm, TR = 2000 ms, TE = 30 ms, flip angle = 80°, volumes acquired = 200. The total time of fMRI scanning was 6 min 40 sec.

### 2.4 Image Preprocessing

The rs-fMRI data processing was conducted using the toolbox for Data Processing & Analysis of Brain Imaging (DPABI, http://rfmri.org/dpabi) [22]. To ensure the quality of the signal, we discarded the first five-time points, and the remaining points underwent slice-time correction. Subsequently, the images were realigned to correct for head motion, and any images in which translation was > 3 mm or rotation > 3° were excluded. The realigned fMRI images were then processed by segmentation of the gray matter (GM), white matter (WM), cerebrospinal fluid (CSF) and normalization to the MNI template. Then, a half-maximum Gaussian kernel (6-mm full width) was used to smooth the images. Finally, band-pass temporal filtering (0.01-0.1 Hz) was used to remove the effects of very-low-frequency drift and high-frequency noise.

### 2.5 GICA Analysis

After preprocessing, we employed a spatial group ICA (version 4.0b; mialab.mrn.org/software/gift/) to decompose the preprocessed fMRI images into different resting-state networks [23]. First, the fMRI data were concatenated by principal component analysis (PCA) and 50 principal components were obtained. Then, the Infomax ICA algorithm [24] was implemented, and the GIG-ICA back reconstruction was used to reconstruct spatial maps of each IC. To estimate the reliability of the spatial aggregated maps, we iterated 20 times in ICASSO implemented in GIFT [25]. Finally, we spatially correlated independent components with established templates (Stanford’s Functional Imaging in Neuropsychiatric Disorders Lab; http://findlab.stanford.edu/functional_ROIs.html), and the independent components will be identified with high correlation (all correlations > r = 0.25) [26]. In this way, 17 ICs were extracted from 50 ICs as described in previous studies: (1) IC should exhibit peak activations in the grey matter; (2) low spatial overlap with known vascular, ventricular, motion, and susceptibility artifacts; (3) should have time courses dominated by low-frequency fluctuations (ratio of powers below 0.1 Hz to 0.15-0.25 Hz in the spectrum) [27].

### 2.6 dFNC Estimation

The dFNC analysis was conducted by using the temporal dynamic FNC toolbox in GIFT. The framework for characterizing dFNC is depicted in Fig. (**1**). The sliding window approach [14] with a size of 30 TRs rectangle window was used to explore the time-varying characters of FC between networks based on the IC time series. We chose the 1 TR as our tapered window steps and convolved with a Gaussian (σ = 3 TRs) to segment the resting-state time series by sliding across the whole 195 TR scans. In the Graphical LASSO framework, we used the 10 repetitions of the L1 norm penalty to promote sparsity in estimations [28]. Finally, we obtained 165 windows and 17 × 17 pairwise FC matrices by regularized precision matrix (inverse covariance matrix) [29]. To improve normality and comparability, we also performed Fisher's Z-transform on the matrix, which can convert the correlation value of the function matrix into a Z-value [30].

To identify recurring functional states, we conducted a k-mean clustering on the dFNC, which was iterated 50 times with the L1 distance (Manhattan distance) function to estimate the similarity between matrices. The elbow method was used to identify the optimal number of clusters ranging from 2 to 6, which was computed as the sum of the square distance error between the particle of the cluster and the sample point in the cluster. In the next 50 clustering iterations, we obtained 4 reoccurring FC states and the 4 highly structured dFNC states that reoccurred throughout individual scans and across participants. To examine the temporal properties of dFNC states, we calculated fractional rate and mean dwell time from the categorized data. The fractional rate represents the proportion of each state. The mean dwell time represents the average duration of time spent in each state, which was calculated by averaging the number of consecutive windows belonging to one state before switching to another.

### 2.7 Dynamic Topologic Analysis

The variability of the topological organization of the functional connectivity network was analyzed by using GRETNA software (www.nitrc.org/projects/gretna). First, we defined those GICA-identified 17 ICNs as nodes, dFNC between pairs of ICNs as edges. And, we constructed a 17×17 connectivity matrix for each subject and each state. Next, we binarized the FC matrices of all windows with a series of sparsity thresholds, where edges larger than the threshold was designated as 1, and 0 when it was smaller than the threshold. Only the positive values of connectivity were considered in this study. Following the previous studies, the sparsity, which is the ratio of the number of existing edges divided by the maximum possible number of edges in a network, was determined. The thresholds ranged from 0.10 to 0.35 (with an interval of 0.05) for further analyses [17, 31].

Then, we calculated both network efficiency and small-world properties in a series of the adjacent matrix for all participants in each state. The four parameters were calculated: (1) global efficiency (Eg), which measures the global efficiency of parallel information transfer in a network and (2) local efficiency (Eloc), which measures how efficient communication is among the first neighbors of a given node when it is removed. (3) cluster efficiency (aCP), which is the area under the curve (AUC) of the clustering coefficient of a network for each subject. (4) path length (aLP) which is the AUC of the shortest path length of a network for each subject. Finally, we applied the area under the curve (AUC) for each network to provide a scalar that does not depend on a specific threshold selection as described in previous studies [31, 32].

### 2.8 Statistical Analysis

The demographic and clinical characteristics between groups were compared with SPSS 20.0 (SPSS Statistics, IBM, Armonk, NY) [33]. To explore temporal and structural properties of dFNC patterns at baseline, we used a two-sample t-test to compare functional efficiency, mean dwell time, fraction rate and global/local efficiency differences between heroin and healthy control group (*P* < 0.05, FDR corrected). Then, paired t-test was employed to assess whether brain recovery would be detected in temporal and structural properties of dFNC patterns in HUs after abstinence (*P* < 0.05, FDR corrected). Finally, we performed correlation analyses to investigate possible relationships between abnormal functional connectivity, temporal and topological properties and clinical variables, including craving and abstinence duration.

## 3 RESULTS

### 3.1 Demographic Characteristics of Participants

Detailed information is displayed in Table **1**. Except for nicotine use and education, there were no significant group differences in age, gender and alcohol use between HUs and HCs (After including education and nicotine as covariates, similar results were observed). Five HUs failed to finish the prolonged abstinence longitudinal study, and only 35 HUs were collected during the second time point (Table **1**).

### 3.2 Identification of ICNs, Clustering Analysis and dFNC Analysis

**Fig. (2):**
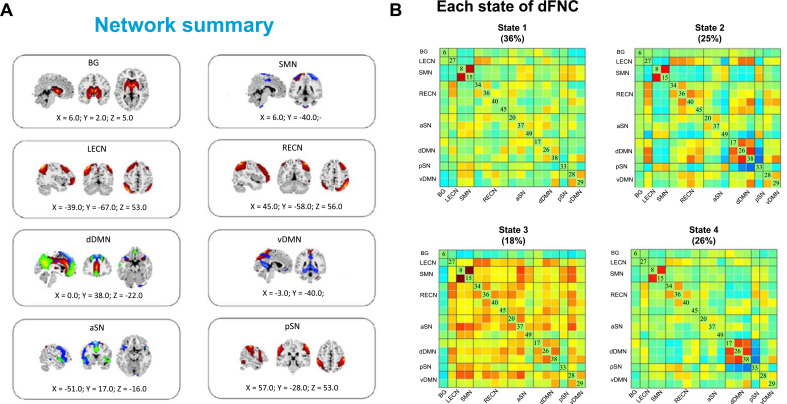
(**A**) 17 ICs were selected and grouped into 8 functional networks: basal ganglia network (BG), anterior salience network (aSN), posterior salience network (pSN), left executive-control network (LECN), right executive-control network (RECN), ventral default-mode network (vDMN), dorsal default-mode network (dDMN) sensorimotor network (SMN); (**B**) Each state of dFNC after k-means clustering in all subjects and resulting in four distinct states.

Based on the GICA result, 17 ICs were assigned to the following eight networks which have been widely studied in heroin addiction (Fig. **2A**). The dFNC among ICNs was calculated, and four clusters were determined by using k-means clustering (Fig. **2B**). The state 1 showed strong dynamic FC between BG and another network and accounted for the largest proportion, which was similar to the static FNC. In contrast, state 2 showed a negative dynamic FC of LECN-SMN/aSN/pSN but positive in state 3. State 2 showed higher dynamic FC of LECN-dDMN and RECN-dDMN compared with state 4. Except for the BG network, most other networks showed positive dynamic FC in state 3. In state 4, higher dynamic FC of pSN-dDMN was observed compared with state 2.

### 3.3 Temporal Properties Reconfiguration of dFNC in State 4

**Fig. (3):**
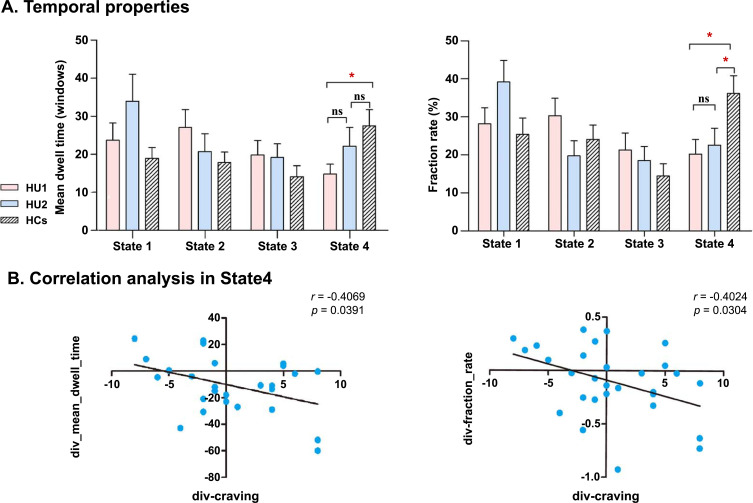
(**A**) Differences in mean dwell time and fraction rate between HCs and HUs and the alteration in mean dwell time and fraction rate in HUs between baseline and after abstinence (we used HU1 and HU2 to represent the HUs at baseline or after abstinence, respectively). Data are presented as mean values ± SEM. (**B**) A negative association was observed between craving changes and fraction rate and mean dwell time changes (between baseline and after prolonged abstinence).

Comparisons of temporal properties indicated that HUs showed less occurrence in state 4 than HCs, which was not found in other states (Mean dwell time: (*t*(78) = 2.550, *P* = 0.013; Fraction rate: (*t*(78) = 2.611, *P* = 0.011). After prolonged abstinence, the mean dwell time and fraction rate of HUs in state 4 increased significantly (Mean dwell time: (*t*(73) = -1.348, *P* = 0.182; Fraction rate: (*t*(73) = -0.394, *P* = 0.695). Unfortunately, the trend of recovery did not survive FDR correction. More importantly, negative correlations were observed between craving scores changes and mean dwell time/ fraction rate changes (mean dwell time change: r = -0.4069, *P* = 0.0391; fraction rate change:r = -0.4020, *P* = 0.0304) (Figs. **3A** and **3B**).

### 3.4 Functional Connectivity Reconfiguration of dFNC in State 4

**Fig. (4):**
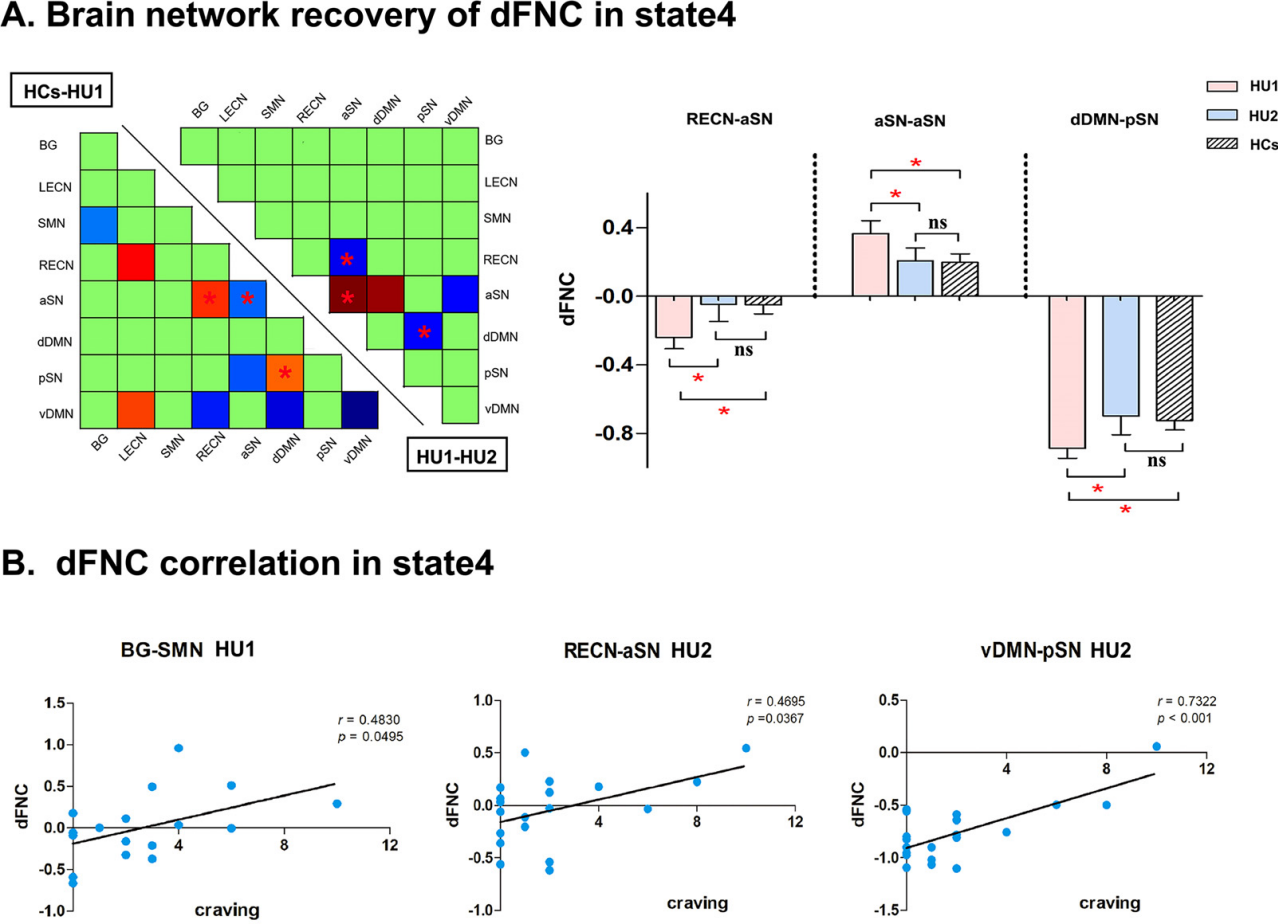
(**A**) Group differences of dFNC in state 4. At baseline, heroin users and healthy controls had significant differences in many brain networks. But after prolonged abstinence, only a few brain networks showed a recovery trend in state 4. (The *marks the brain network with recovery trend, *P* < 0.05). The bar graph showed the actual FC strength among the three groups in brain networks which showed a recovery trend. (**B**) Correlation analysis. The BG-SMN dynamic FC strength of state 4 was correlated with craving at baseline. After abstinence, the RECN-aSN and vDMN-pSN dynamic FC strengths had a significant positive correlation with craving. We used HU1 and HU2, which represent baseline and after prolonged abstinence, respectively.

At baseline, the HUs showed significantly higher connectivity in RECN-aSN, (*t*(53) = 2.272, *P* = 0.027) dDMN-pSN(*t*(53) = 2.050, *P* = 0.045) and aSN-aSN(*t*(53) = -2.122, *P* = 0.039) compared with HCs in state 4. After prolonged abstinence, the recovery trend of the connectivity between these brain networks was observed (RECN-aSN: (*t*(9) = -2.857, *P* = 0.019), dDMN-pSN: (*t*(9) = 2.753, *P* = 0.022) and aSN-aSN(*t*(9) = 3.523, *P* = 0.006)) (Fig. **4A**). At baseline, a positive association was found between craving scores and the connectivity of BG-SMN in state 4 (r = 0.4830, *P* = 0.0495). After prolonged abstinence, the connectivity of RECN-aSN and vDMN-pSN were both correlated with craving scores (RECN-aSN: r = 0.4695, *P* = 0.0367; vDMN-pSN: r = 0.7322, *P* < 0.001) (Fig. **4B**).

### 3.5 Dynamic Topological Properties Reconfiguration in State 4

**Fig. (5):**
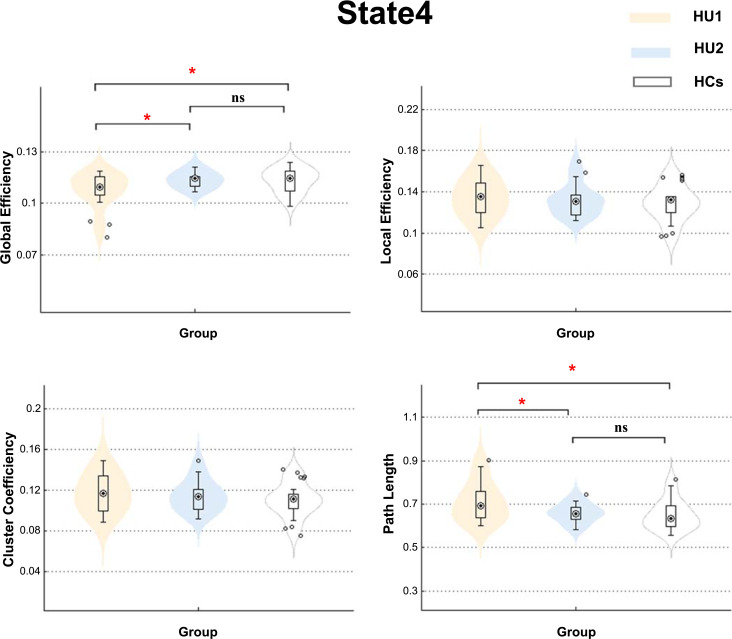
Dynamic topological properties. Global efficiency, local efficiency, cluster efficiency, and path length in the different states are shown using violin plots for different groups (HU1, HU2, and HCs).

At baseline, HUs showed lower global efficiency and higher path length (*t*(53) = 2.293, *P* = 0.026 for Eg and *t*(53) = -2.627, *P* = 0.012 for aLP) in state 4. After prolonged abstinence, the lower global efficiency and higher path length in state 4 showed a potential recovery trend (*t*(36) = -2.365, *P* = 0.025 for Eg and (*t*(36) = 2.294, *P* = 0.028 for aLP) and are now comparable to HCs’ performance (*t*(41) = -0.037, *P* = 0.971 for Eg and (*t*(41) = -0.478, *P* = 0.636 for aLP). However, a similar phenomenon was not detected for local efficiency or cluster coefficient (Fig. **5**).

## 4 DISCUSSION

The human brain is a complex patchwork that needs functional interactions of large-scale brain networks [1]. Disrupted coupling of large-scale networks (*e.g.,* SN, DMN, ECN) had been found to be associated with craving and relapse behavior in heroin users. Partial regional and circuit level recovery was also found in substance use disorders, including heroin, after prolonged abstinence [8, 10, 34-37]. However, until recently, the above works only focused on the aberrant patterns in static RSFC, which failed to reveal time-varying characteristics of neural mechanisms/pathophysiology in addiction. In the current study, we identified four occurrence states by conducting a dFNC analysis. Among them, state 4 was characterized by the strong dFNC strength between SN and other networks. In HUs, the lower mean dwell time and fewer fraction rate in state 4, as well as the SN centered dFNC with ECN and DMN, were partially normalized after prolonged abstinence. Our results provide direct evidence that prolonged abstinence can induce reconfigurations of SN with ECN and DMN, which might explain the neural mechanisms of the reduced craving in HUs.

### 4.1 The Reconfigurations of dFNC in HUs After Abstinence

The state 4 was represented by strong dFNC strength between SN and other networks, mainly in ECN and DMN. In the current study, our results showed that HUs tended to spend lower mean dwell time and fraction rate in this state. Furthermore, after prolonged abstinence, the mean dwell time and fraction rate in state 4 were increased in HUs. Our correlation analysis revealed that state 4’s occurrence changes showed a significant negative correlation with craving changes (Fig. **3**). Emerging evidence suggests that SN is an integral hub that may play an important role in mediating dynamic interactions between other large-scale brain networks involved in externally oriented attention (*i.e.,* ECN) and internally oriented self-related mental processes (*i.e.,* DMN) [12, 38]. The results, *i.e*., more occurrence in state 4 after abstinence, might be associated with the reduced craving in HUs due to the more involvement of SN.

The implications of SN, ECN and DMN had been revealed separately in HUs. In detail, the reduced RSFC between sub-regions of SN was found in HUs [39]. And higher activation in SN with increasing craving when confronted with heroin-related cues, suggesting that the abnormal in SN may be related to the high vulnerability of relapse in short-term abstinent heroin-dependent subjects [20]. The degradation of ECN was also reported in HUs. Furthermore, significant activation of the DLPFC within ECN was observed when confronted with heroin cues [7]. The lack of regulation by dopamine in ECN underlies the enhanced motivational value of drugs in their behavior and the loss of control over drug intake [4]. Decreased FC within DMN was also detected in HUs compared with HCs [40-42]. Previous studies focused on the static RSFC rather than the dFNC. The interactions among ICNs (*e.g.,* SN, ECN, DMN and BG) in HUs remained unclear; less is known about the reconfigurations after prolonged abstinence. Our findings filled this gap by demonstrating that hyper-dFNC of SN with DMN and ECN in state 4 was partially reversed after prolonged abstinence, which was similar with dFNC patterns in HCs. The results in the current study were also in line with the previous cocaine study; the lower static RSFC of SN-ECN was enhanced after 3-6 months of abstinence [35]. Furthermore, at baseline, the craving was positively correlated with dFNC of BG-SMN in state 4 [43, 44]. After prolonged abstinence, the craving was correlated with the dFNC of the SN with ECN and DMN (Fig. **4**). Our correlations analysis revealed more interventions of SN over DMN and ECN with craving. Thus, the reconfigurations of dFNC among SN, ECN and DMN might be the underlying mechanisms of reduced craving after prolonged abstinence in HUs.

### 4.2 The Improved Global Efficiency in HUs after Abstinence

It had been reported that heroin-dependent individuals showed abnormal global efficiency and path length in brain white matter structural networks [45]. Similar abnormal phenomena were also found in the resting-state connectivity network [46]. Above findings from the structural and functional perspective suggested the altered topology properties may reflect the altered robustness of global information processing. The current study conducted dFNC analysis found the state 4 may play an important role in revealing heroin addiction pathophysiology which further supported by reduced efficiency of global information transfer in functional brain networks. At baseline, the global efficiency is lower, and the path length is longer than HCs, identifying an expansive disconnected subnetwork in HUs. But after prolonged abstinence, our study observed the global dysfunction of multisensory information processing and integration could be normalized in HUs (Fig. **5**). These findings enhanced our understanding of the underlying pathophysiology of heroin addiction and suggested that not only the temporal and dynamic functional connectivity between networks have recovered, but also the properties of topologic organizations have developed towards HCs.

However, there are several limitations to this study. First, the sample size of the current study is small. Among 40 participants, 5 patients failed to complete the follow-up study, and the different sample size of patients and controls was also a limitation. Second, in our study, we did not rule out the confounding effect of vigilance which may influence the dFNC during MRI scans. Thus, future studies could include an independent dataset to validate our findings, and future studies could include EEG, cardiac, respiratory, and independent datasets to monitor vigilance levels during the MRI scan. Third, regression analysis did not survive multiple comparisons correction. Further study with larger sample size is necessary to solve this problem. Fourthly, in light of evolving standards in the field, the length of our resting-state scan is relatively short. Therefore, a longer resting-state scan should be used in future studies. Finally, our study used functional neuroimaging, which identified brain regions in which activity is correlated with behaviors or symptoms. Despite their advantages, our technology only revealed correlations, not causation. Therefore, a more advanced method is needed in future studies to explain the heterogeneous causal findings.

## CONCLUSION

In conclusion, our longitudinal follow-up study observed the large-scale brain network reconfiguration from the dynamic perspective in HUs after prolonged abstinence and improved the understanding of the neurobiology of prolonged heroin abstinence. Then, it also provides a new target for the treatment of addiction in the future. It is speculated that prolonged abstinence could reconfigure the inappropriate signal of SN and increase global integration in the brain of HUs.

## Figures and Tables

**Fig. (1):**
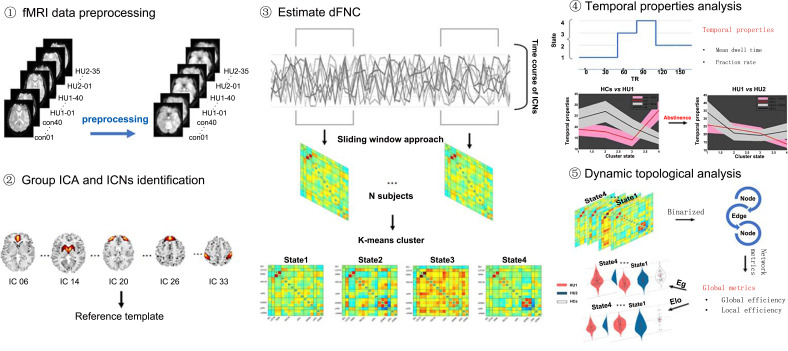
The framework of dynamic functional network connectivity (dFNC) state analysis and dynamic topological/temporal properties analysis; **①** Preprocessing the raw fMRI data; **②** Group independent component analysis (GICA) was used to select 17 ICs and assigned into intrinsic connectivity networks (ICNs); **③** Based on sliding-window approach, time-coursed of each individual was segmented into 165 windows (window size was 30 TR). Then, FC matrices of all subjects were clustered using k-means algorithm and four dFNC states were obtained; **④** Two temporal properties, including fraction rate and mean dwell time, were calculated for all participants in different time scale; **⑤** In dynamic graph theory analysis, global topological properties were computed across all state and all participants in different time scale.

**Table 1:** Demographic and clinical characteristics of participates.

**Group**	**HU**	**HC**	**t/χ^2^**	***p*-value**
Age (years)	41.60 ± 6.43	40.80 ± 9.47	0.442	0.660
Gender	28/12	26/14	0.228	0.812
Alcohol (AUDIT)	1.98 ± 4.08	2.85 ± 4.53	-0.908	0.367
Nicotine (FTND)	5.20 ± 2.50	3.15 ± 2.57	3.616	0.001
Education (years)	8.45 ± 3.16	10.66 ± 2.39	3.531	0.001
Withdrawal time (days)	40 ± 43.5	-	-	-
Duration (years)	14.72 ± 7.93	-	-	-
Dose of heroin use (g per day)	0.47 ± 0.41	-	-	-
Handiness (R/L)	R	R	-	-
Craving(scores) (baseline)	3.87 ± 2.46	-	-	-
Craving(scores) (289-day prolonged abstinence)	2.23 ± 2.70	-	-	-

**Note:** Unless otherwise specified, data are means ± standard deviation.

## Data Availability

Not applicable.

## LIST OF ABBREVIATIONS

AUC: Area Under the Curve
CSF: Cerebrospinal Fluid
DMN: Default Mode Network
GM: Gray Matter
HCs: Healthy Controls
HUs: Heroin Users
PCA: Principal Component Analysis
SMN: Sensorimotor Network
SN: Saliency Network
WM: White Matter
